# CADASIL in Arabs: clinical and genetic findings

**DOI:** 10.1186/1471-2350-8-67

**Published:** 2007-11-09

**Authors:** Saeed Bohlega, Asmahan Al Shubili, Abdulrahman Edris, Abdulrahman Alreshaid, Thamer AlKhairallah, M Walid AlSous, Samir Farah, Khaled K Abu-Amero

**Affiliations:** 1 Department of Neurosciences, King Faisal Specialist Hospital & Research Centre, Riyadh, Saudi Arabia; 2 Neurology Department, Ibn Sina Hospital, Kuwait; 3 Hera Hospital, Mecca, Saudi Arabia; 4 Islamic Hospital, Amman, Jordan; 5 Department of Genetics, King Faisal Specialist Hospital & Research Centre, Riyadh, Saudi Arabia

## Abstract

**Background:**

Cerebral autosomal dominant arteriopathy with subcortical infarcts and leukoencephalopathy (CADASIL) is increasingly recognized as an inherited arterial disease leading to a step-wise decline and eventually to dementia. CADASIL is caused by mutations in *NOTCH3 *epidermal growth factor-like repeat that maps to chromosome 19. CADASIL cases have been identified in most countries of Western and Central Europe, the Americas, Japan, Australia, the Caribbean, South America, Tanzania, Turkey, South Africa and Southeast Asia, but not in Arabs.

**Methods:**

We studied three families from Saudi Arabia (Family A), Kuwait (Family B) and Yemen (Family C) with 19 individuals affected by CADASIL.

**Results:**

The mean age of onset was 31 ± 6 and the clinical presentation included stroke in 68%, subcortical dementia in 17% and asymptomatic leukoariosis detected by MRI in 15%. Migraine and depression were frequently associated, 38% and 68% respectively. The mean age of death was 56 ± 11. All *NOTCH3 *exons were screened for mutations, which revealed the presence of previously reported mutations c.406C>T (p.Arg110>Cys) in two families (family A&B) and c.475C>T (p.Arg133>Cys) mutation in family C.

**Conclusion:**

CADASIL occurs in Arabs, with clinical phenotype and genotype similar to that in other ethnic groups.

## Background

Cerebral autosomal dominant arteriopathy with subcortical infarct and leukoencephalopathy (CADASIL) is becoming the most common form of adult onset hereditary syndrome characterized by recurrent transient ischemic attacks (TIA) and strokes, leading to progressive dementia, migraine with aura and psychiatric disturbances [1-3]. Symmetrical white matter abnormalities are invariably seen, while small subcortical infarcts are often reported [4]. The extent of the MRI lesions increases with age. Atypical anteriopathy with electron-dense granular deposition in the media of small cerebral arteries underlies the pathology of this disorder [5]. This initial thickening and expansion of the extracellular matrix can be found to a lesser extent in extracerebral arteries, such as skin arterioles [6]. Mutation in the *NOTCH3 *gene is usually linked to CADASIL [1]. *NOTCH3 *encodes a 300-kd transmembrane protein with a receptor and cell signal transduction function. This receptor is expressed in vascular smooth muscle cells [7]. Mutations are almost always mis-sense mutations causing the loss or gain of a cysteine residue and are detected in over 90% of patients [8,9]. All mutations were located within the epidermal growth factor (EGF) repeats in the extracellular domain of the *NOTCH3 *gene and a strong clustering of the mutations were observed in exons 3 and 4 [8-10]. Thus, there are more than 81 *NOTCH3 *mutations reported on the human genome mutation database [11] to-date. Therefore, CADASIL is far more common than previously perceived and it may often be misdiagnosed because it can present under various guises [10].

CADASIL cases have been identified in most countries of Western and Central Europe, the Americas, Japan, Australia, Tanzania, South America, the Caribbean, Turkey [12], South Africa [6] and Southeast Asia [13-16]. To our knowledge, there are no published reports of CADASIL in native Arabs yet. Here we describe the clinical and genetic findings in three families from various Arab countries.

## Methods

### Family enrollment

Requests to identify cases and participants in this study was done through mailing a brief Inclusion Criteria form (see below) for the disease to all members of the Pan Arab Union of Neurological Sciences. Families were included when an index case had both a history of transient ischemic attacks (TIA) or subcortical stroke of unidentified etiology, positive family history of stroke with early death or dementia compatible with autosomal dominant traits, and a cranial MRI scan showing diffuse or focal microangiopathic white matter abnormalities. Three families with pure ethnic background fulfilled the inclusion criteria. Family (A) from Saudi Arabia, family (B) was from Kuwait and family (C) was from Yemen. Affected individuals, their parents (if alive) and siblings were studied in these families. This research followed the tenets of the Declaration of Helsinki. All participants signed an informed written consent, which was approved by the King Faisal Specialist Hospital-Institutional Review Board KFSH-IRB. The approved informed consent included an approval for inclusion of family members in this study and for publication of findings.

### Clinical assessment

**Figure 1:**
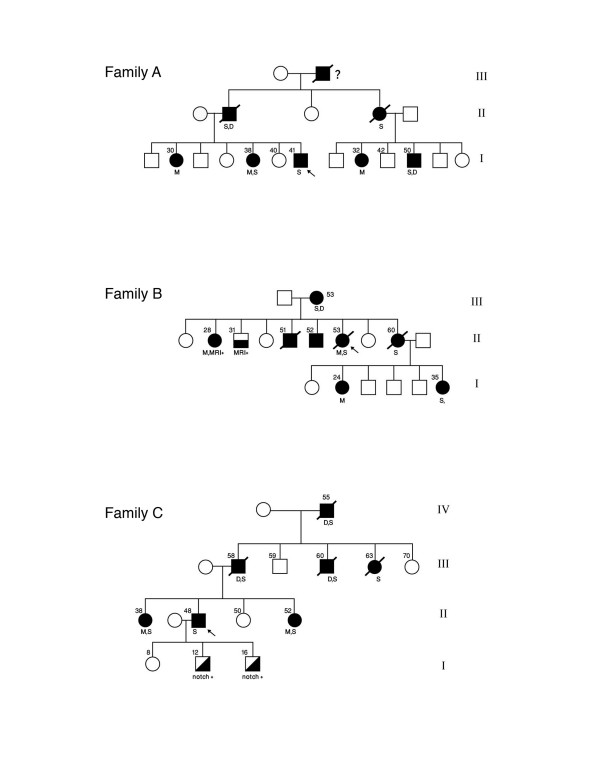
Pedigree of the three Arab studied kindreds. S: stroke, D: dementia, M: migraine, P: psychiatric manifestations. Number(s) at the top indicates age at death or at time of examination. Diagonal fill indicates asymptomatic positive for mutations. Horizontal fill indicates asymptomatic positive with abnormal brain MRI.

All subjects, their siblings and other available family members (Figure 1) have had detailed clinical evaluations and assessment of risk factors. Previous and current imaging results were collected. In one family MRI brain scans were performed in all sib ship of the index case (Family B). Vascular dementia was diagnosed according to the "NINDS-AIREN Criteria for the diagnosis of vascular dementia" [17]. In summary, probably vascular dementia includes: memory impairment, cognitive decline in one or more domains, and presence of focal signs on neurological examination (gait problems, personality and mood change and pseudobulbar involvement).

### Sample collection and DNA extractions

Five ml of peripheral blood were collected in EDTA tubes from all participating individuals after obtaining their written informed consent. DNA was extracted from whole blood samples of all CADASIL patients and their family members using the PURGENE DNA isolation kit from Gentra Systems (Minneapolis, USA).

### Mutation analysis of the NOTCH3 gene

DNA from patients and their families was amplified using primers designed to amplify the 33 exons, including the intron-exon boundaries, of the *NOTCH3 *gene. The same amplification primers were also used for sequencing. Sequencing was carried out using the Dynamic Terminator Reagent Sequencing kit [Amersham Pharamcia, US]. The samples were then run on the DNA analyzer [MegaBACE 1000 Capillary system; Molecular Dynamics, Amersham Pharmacia Biotech]. Data from the analyzer were analyzed using the Chromas-Pro version 1.34 (Technelysium, Pty, Ltd, Australia). We also investigated the frequency of each detected mutation in our normal controls (see Table 1). In this study, we excluded known proven silent-polymorphisms found in patients and controls from reporting and further analysis.

**Table 1:** Summary of the NOTCH3 mutations detected in our CADASIL families

**Family**	**Ethnicity**	**Mutation detected**	**Exon**	**Amino acid change**	**Domain**	**Frequency in our controls (n= 100)**
A	Saudi	c.406C>T	3	p.Arg110>Cys	EGF2	ND
B	Kuwait	c.406C>T	3	p.Arg110>Cys	EGF2	ND
C	Yemen	c.475 C>T	4	p.Arg133>Cys	EGF3	ND

EGF = Epidermal Growth Factor Like Repeats.

ND = not detected

## Results

**Figure 2:**
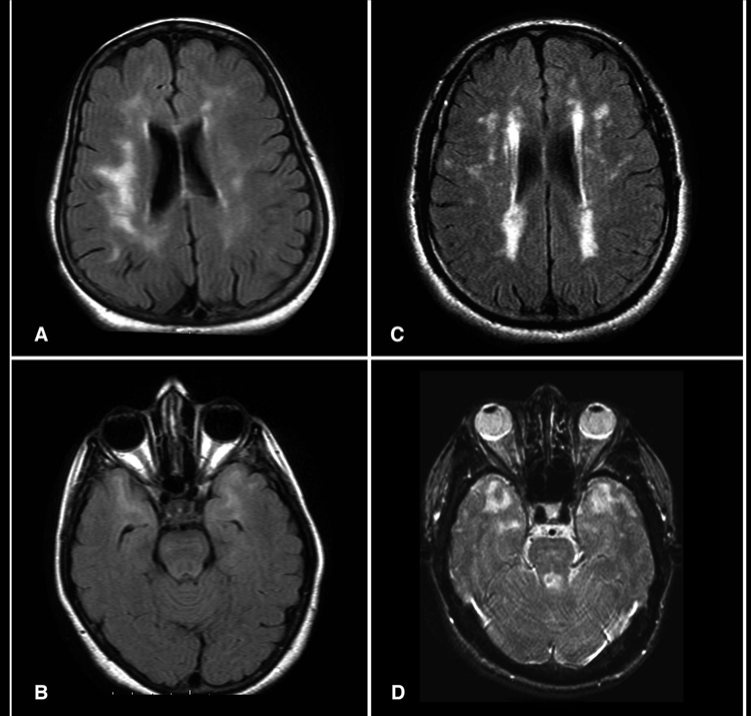
Axial FLAIR (a, b & c) and T2 weighted (d) Brain MRI from patients. The exams in 2a and 2b are from asymptomatic patients with depression. Note temporal lobe lesions even in asymptomatic patients (2b). In Figure 2a and d periventricular diffuse white matter ischemic lesion and multiple lacunar lesions in thalamus, pons and basal ganglia.

Nineteen (19) affected individuals, 9 males & 10 females, were examined by at least one of the authors. The mean age of onset was 31 ± 6 years. Stroke or TIA were noted in 68% of cases, the majority of episodes led to motor syndrome (hemiparesis, pseudobulbar palsy, dysphagia and incontinence) followed by sensory deficit and ataxia. 22% of examined patients had prominent cognitive decline and met the diagnostic criteria for vascular dementia [17] with stepwise deterioration, gait problems and urinary incontinence. Depression was the prominent affective disorder (25%) and in few patients was the only presenting symptom. Age of death was retrospectively identified and the mean age of death was 56 ± 11 years in the 8 patients studied. Migraine with aura was the predominant feature in members of Family B and affected members had histories of at least one vascular headache attack starting in the early 20s, followed by TIA or stroke 8 – 12 years later. One individual with depression and one with psychosis and ischemic changes in their MRIs (Figure 2a, 2b). Interfamilial and intra-familial variability with regards to presenting symptoms and of age at death was noted in most of the participants.

The following is the clinical and molecular findings summary of the three (3) families studied.

### Family A

The proband was examined at age 40 because of sudden left-sided weakness. He has a history suggestive of multiple attacks of TIA at age 37 and 38, and an episode of right sided numbness at age 39. The proband father was reported to have migraine attacks at age 33 and repeated stroke starting at age 40. He died at age 58. His aunt had stroke at age 40 and died at age 51 with dementing illness. His sister had one episode of stroke at age 38, his brother had multiple minor strokes, showed evidence of pseudobulbar affects, and bilateral pyramidal findings leading to abnormal gait at age 36. MRI brain for proband, his father, sister and brother showed multiple ischemic lesions bilaterally with marked brain atrophy in the father. Sequencing of the *NOTCH3 *gene, revealed the presence of heterozygous c.406C>T (p.Arg110>Cys) pathogenic mutation in exon 3 of the *NOTCH3 *gene. This mutation was previously described [1] and was not detected in 100 controls with matching ethnicity.

### Family B

In this Kuwaiti family the proband had migraine in the mid-20s, and multiple strokes were noted at age 28. The mother had migraine and stroke in her early 30s and died at age 53. Six out of eight siblings were affected; two were asymptomatic with white matter disease on MRI, three died at age 51, 53 and 60 respectively, with a history of migraine, stroke and dementia. One sister with multiple strokes died at age 51. None had hypertension or diabetes and all blood tests and cardiac evaluations were normal. The older sister had one daughter with migraine and stroke starting at age 30, while the other had experienced migraine with aura at age 21. Sequence analysis of the *NOTCH3 *gene; revealed the presence of heterozygous c.406C>T (p.Arg110>Cys) pathogenic mutation in exon 3. This is a previously reported mutation that was not found in 100 controls of matching ethnicity.

### Family C

A 48-year old man from southern Yemen, known to have rheumatoid arthritis was seen with a five-month history of repeated attacks of numbness right side. He has three other sisters. A Sister aged 52 had a long-standing history of migraine, another had multiple strokes starting at age 36. The father died at age 58 because of dementia and repeated stroke. An uncle died at age 60 after a 12-year history of stroke and vascular dementia, while an aunt died at age 63 with accelerated stroke over four years. MRI from the proband and his sister showed multiple ischemic white matter lesions (Figure 2c, 2d). Sequencing the *NOTCH3 *gene revealed the presence of a previously known heterozygous mutation c.475C>T (p.Arg133>Cys) in exon 4 [1]. This mutation was not found in our controls. Table 1 summarizes the molecular findings in all families.

## Discussion

The families described in this report showed various characteristics that are typical of CADASIL, an autosomal dominant syndrome, with migraine, recurrent ischemic strokes, cognitive impairment, depressive disorder and premature death within 10 – 20 years after onset [4]. CADASIL affects young and old adults, irrespective of vascular risk factors. In two of our families the affected sibling of probands had migraine with aura in their early 20s, followed by recurrent ischemic attacks in their mid 30s. Young onset of this syndrome had been reported previously [12,16]. The clinical course of the disease is heterogeneous, even within the same family as noted previously [1,5] (Figure 1).

MRI findings in our families include focal lacunar infarct and diffuse T2 weighted hyperintensity, usually including the temporal lobes and external capsules (Figure 2b, 2d). Positive MRI findings in asymptomatic individuals or in-patients who presented with depression or a psychiatric problem (Family B) indicated that *NOTCH3 *gene signalling may start early and may have different clinical presentations. The reported penetrance of MRI abnormalities is usually complete after age 35 and the mean age of clinical onset was 45 ± 10 years [3,4].

*NOTCH3 *mutations have been identified as a genetic cause of CADASIL. Numerous mutations in this gene have been described in recent years along with genetic heterogeneity of the disease [9]; however, until now no genotype-phenotype relationship has been described [9].

The molecular findings in our families are in agreement with previous studies in other populations from different ethnic groups. All reports have found exon 4 to be the most common site of mutation and exon 3 to be the second most common site [7,8,18].

CADASIL has been reported across various ethnic groups, with predominance among the French, German and Italian populations [2,6]. It has seldom been reported from Asia [14]. Families from Japan [6], Korea [13], Taiwan [14], Turkey [12] and Thailand [15] have also been reported.

## Conclusion

We conclude that CADASIL can be seen in Arabs, and is probably under-diagnosed with similar clinical and genetic presentation as seen in other ethnic groups.

## Abbreviations

CADASIL Cerebral autosomal dominant arteriopathy with subcortical infarct and leukoencephalopathy

EDTA Ethylene Diamine Tetraacetic Acid

EGF Epidermal Growth Factor

MRI Magnetic Resonance Imaging

PCR Polymerase Chain Reaction

TIA Transient Ischemic Attacks

## Competing interests

The author(s) declare that they have no competing interests.

## Authors' contributions

SB, AS, AE, AA, TK, WS and SF were all involved in diagnosing the cases and carrying out the clinical evaluations. SB was in charge of recruiting patients and writing the clinical part of the manuscript. KAA performed the mutation analysis and laboratory work associated with it and writing the genetic part of this manuscript. All authors have read and approved the final manuscript.

## Pre-publication history

The pre-publication history for this paper can be accessed here:


